# Novel therapeutic potential of angiotensin receptor 1 blockade in a rat model of diabetes-associated depression parallels altered BDNF signalling

**DOI:** 10.1007/s00125-019-4888-z

**Published:** 2019-05-03

**Authors:** Lilla Lenart, Dora B. Balogh, Nikolett Lenart, Adrienn Barczi, Adam Hosszu, Tamas Farkas, Judit Hodrea, Attila J. Szabo, Krisztian Szigeti, Adam Denes, Andrea Fekete

**Affiliations:** 1 1st Department of Pediatrics, Semmelweis University, Bókay János u. 53-54, Budapest, 1083 Hungary; 2 MTA-SE Lendület Diabetes Research Group, Budapest, Hungary; 3 “Momentum” Laboratory of Neuroimmunology, IEM HAS, Szigony u. 43, Budapest, 1083 Hungary; 4 Progressio Ltd, Budapest, Hungary; 5 MTA-SE Pediatrics and Nephrology Research Group, Budapest, Hungary; 6 Department of Biophysics and Radiation Biology, Semmelweis University, Budapest, Hungary

**Keywords:** Angiotensin receptor 1 blocker, Brain-derived neurotrophic factor, Depression, Neuroinflammation, Renin–angiotensin–aldosterone system

## Abstract

**Aims/hypothesis:**

Diabetes is a worldwide epidemic linked with diverse diseases of the nervous system, including depression. A few studies suggested a connection between renin–angiotensin–aldosterone system blockers and reduced depressive symptoms, although underlying mechanisms are unclear. Here we investigated the antidepressant effect and the mechanisms of action of the angiotensin receptor 1 blocker (ARB) losartan in an experiential model of diabetes-associated depression.

**Methods:**

Experimental diabetes was induced by streptozotocin in adult male Wistar rats. After 5 weeks of diabetes, rats were treated for 2 weeks with a non-pressor oral dose of losartan (20 mg/kg). In protocol 1, cerebrovascular perfusion and glial activation were evaluated by single-photon emission computed tomography–MRI and immunohistochemistry. In protocol 2, behaviour studies were performed (forced swim test and open field test). Hippocampal proinflammatory response and brain-derived neurotrophic factor (BDNF) signalling were also assessed.

**Results:**

Here, we show that diabetic rats exhibit depression-like behaviour, which can be therapeutically reversed by losartan. This action of losartan occurs via changes in diabetes-induced neuroinflammatory responses rather than altered cerebral perfusion. We also show that as a part of its protective effect losartan restores BDNF production in astrocytes and facilitates BDNF–tropomyosin receptor kinase B–cAMP response element-binding protein signalling in the diabetic brain.

**Conclusions/interpretation:**

We identified a novel effect of losartan in the nervous system that may be implemented to alleviate symptoms of diabetes-associated depression. These findings explore a new therapeutic horizon for ARBs as possible antidepressants and suggest that BDNF could be a target of future drug development in diabetes-induced complications.

**Electronic supplementary material:**

The online version of this article (10.1007/s00125-019-4888-z) contains peer-reviewed but unedited supplementary material, which is available to authorised users.

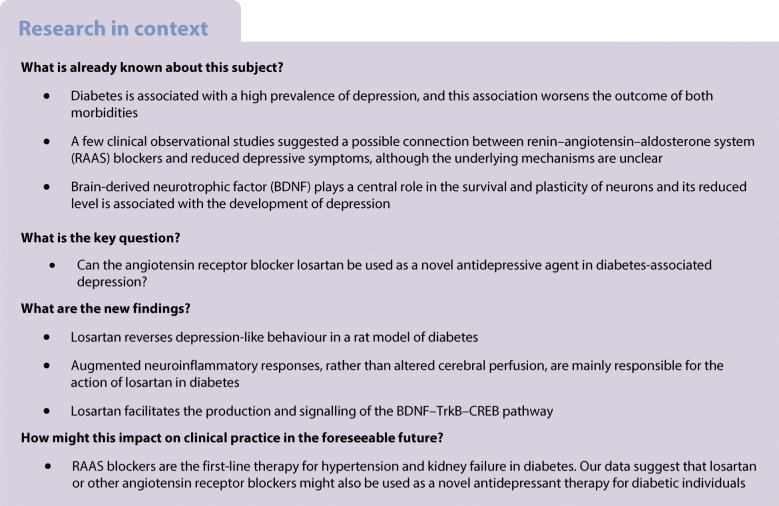



## Introduction

Noncommunicable diseases represent a major challenge for health systems, further augmented by the co-occurrence of multiple chronic conditions. While both diabetes and depression present huge socioeconomic problems affecting millions of people worldwide, the role of diabetes in increasing the risk of depression is only beginning to emerge [1].

The link between the two diseases is unclear at present due to lack of appropriate mechanistic insight. Depression causes deterioration in carbohydrate metabolism and increases the frequency of complications; it also impairs the quality of life and reduces life expectancy [2]. In addition, the incidence of depression is markedly increased in individuals with diabetes [3]. Since diabetes is now widely recognised as a state of chronic systemic inflammation [4], inflammatory actions could represent a plausible mechanism through which diabetes and depression interact [5]. However, there is insufficient experimental evidence to support this hypothesis and effective therapies are lacking.

A clear association between depressive symptoms and reduced levels of the growth factor, brain-derived neurotrophic factor (BDNF), has long been recognised [6]. Hippocampal biopsies of individuals with major depression revealed lower levels of BDNF and its receptor, tropomyosin receptor kinase B (TrkB) [7]. Moreover, TrkB signalling has been implicated in the action of antidepressants, even positioning this pathway as a potential predictor for the efficacy of antidepressants [8].

BDNF is predominantly produced in the central nervous system in the form of a precursor (proBDNF) from which mature BDNF (mBDNF) is derived via proteolytic cleavage. mBDNF induces axon growth and synaptic activity and facilitates cell survival through its receptor, TrkB. ProBDNF is itself also biologically active, although it has an opposing function whereby it decreases synaptic activity and activates apoptotic pathways via neurotrophin receptor p75 (p75^NTR^) [9].

Several in vitro and in vivo studies indicate that neuroinflammatory processes, which are widely linked with the development of depression, affect BDNF expression [10]. Notably, lipopolysaccharide or proinflammatory cytokine administration has been demonstrated to remarkably reduce mRNA and protein levels of BDNF in the brain [11, 12].

Renin–angiotensin–aldosterone system (RAAS) inhibitors are currently one of the primary options in the treatment of diabetes and related complications [13]. Recent findings from a large cohort of individuals with type 1 diabetes showed that RAAS-modifying medication was associated with a reduced requirement for antidepressants [14]. Furthermore, treatment with the angiotensin receptor 1 blocker (ARB) candesartan significantly improved interpersonal sensitivity and depression scores of diabetic individuals [15].

A local RAAS, expressing all classical signalling pathways, also exists in the brain, where it regulates a wide variety of biological functions including blood pressure, body temperature, memory, behaviour and learning [16]. Clinical studies have confirmed that increased RAAS activity in the brain is associated with the development of depression, Alzheimer’s disease and Parkinson’s disease [17–19].

We hypothesised that blockade of RAAS activity by losartan, a widely used treatment regimen in the clinic, may exert antidepressant effects in an experimental model of diabetes. Here we investigated the importance of BDNF signalling in the inflammatory, survival and apoptotic processes of diabetes-associated depression, and suggest novel targets for clinical therapy.

## Methods

### Animals

All experiments were conducted in accordance with guidelines of the Council on Animal Care of the National Health Institution of Hungary (PEI/001/380-4/2013).

Adult, male Wistar rats (Crl:WI; Toxi-Coop, Budapest, Hungary) were housed in temperature- and humidity-controlled units (22 ± 2°C and 70 ± 10%, respectively) with a 12 h light–dark cycle (lights on at 08:00 hours). Three rats (randomly chosen from treatment groups) were housed per cage and had free access to standard chow and tap water. General anaesthesia was induced by inhalation of isoflurane (3% vol./vol.) mixed with air (1 l/min) in an isoflurane vaporiser (Eickemeyer Veterinary Equipment, Twickenham, UK).

### Induction of diabetes and experimental design

**Fig. 1:**
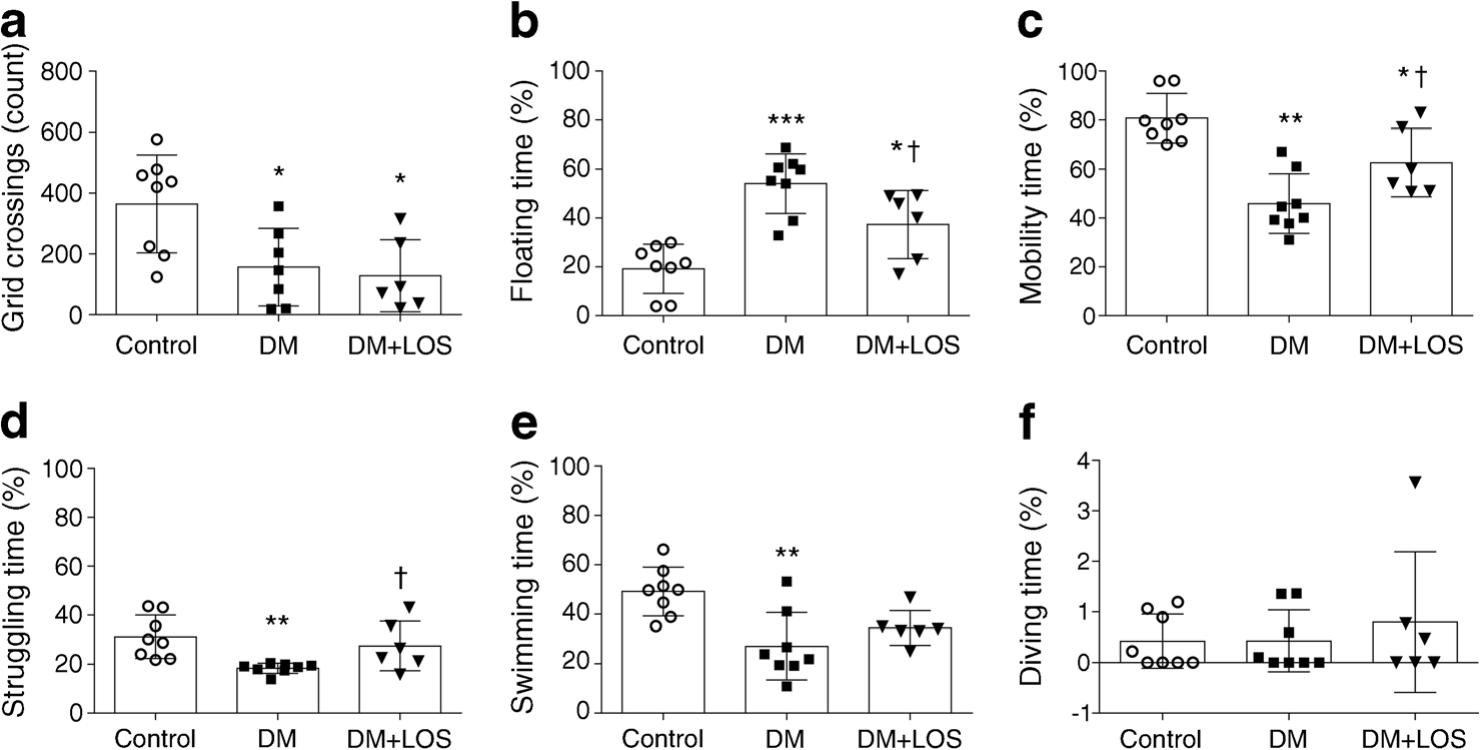
Losartan attenuates depressive behaviour of diabetic rats. OFT and FST were performed on control (non-diabetic) rats, vehicle-treated diabetic rats (DM) and losartan-treated diabetic rats (DM+LOS). (**a**) In OFT, locomotor activity is represented by number of grid crossings. (**b**–**f**) In FST, floating reflects immobility (**b**), while time spent in active mobility (**c**) is represented by struggling (**d**), swimming (**e**) and diving (**f**). Each moving pattern is represented as percentage of time. Data are presented as mean ± SD (*n* = 6–8/group). *p < 0.05, **p < 0.01 and ***p < 0.001 vs control rats; ^†^*p* < 0.05 vs DM

We used streptozotocin (STZ, 65 mg/kg i.p single injection; Sigma-Aldrich, St Louis, MO, USA) to induce diabetes as this is the most widely accepted model of diabetes-associated depression. After 5 weeks of diabetes, rats were randomly divided into groups and treated daily for 2 weeks by oral gavage as follows: (1) isotonic saline (NaCl 154 mmol/l) as vehicle (*n* = 5–7 in protocol 1, *n* = 8 in protocol 2) or (2) losartan (losartan 20 mg/day, dissolved in isotonic saline, *n* = 5 or 6 in protocol 1, *n* = 6 in protocol 2). Experimental protocols are summarised in electronic supplementary material (ESM) Fig. 1.

In protocol 1 imaging studies were conducted at the end of the treatment period. Under terminal anaesthesia, rats were perfused with 4% wt/vol. paraformaldehyde. Brains were post-fixed in a solution containing 4% wt/vol. paraformaldehyde and 10% sucrose at 4°C for 24 h and cryoprotected in 10% wt/vol. sucrose solution.

In protocol 2, behaviour studies and biochemical and molecular measurements were performed at the end of the treatment period. Rats were killed, blood and brains were collected and hippocampi dissected.

In both protocols, age-matched non-diabetic control rats received a single equivalent volume of citrate buffer without STZ and were treated with saline by oral gavage daily for 2 weeks at the same time as the diabetic rats (*n* = 4 or 5 in protocol 1, *n* = 8 in protocol 2). No adverse events were recorded in any of the procedures.

### Measurement of arterial blood pressure and metabolic variables

Systolic and diastolic blood pressure was measured on the tail vein using a CODA Standard monitor system (EMKA Technologies, Paris, France). Mean arterial pressure (MAP) was calculated. In line with the literature, we chose a treatment protocol that avoided blood pressure changes but remained effective in blocking angiotensin II receptor 1. The protocol was successfully applied in several independent experiments in our previous work, wherein changes in MAP never occurred in normotensive Wistar rats following administration of either streptozotocin or losartan (20 mg/kg for 2 weeks) [20]. Blood glucose and fructosamine were photometrically determined from sera on a Hitachi 912 photometric chemistry analyser (Roche Hitachi, Basel, Switzerland).

### Behavioural tests

All behavioural tests were conducted at 08:30 hours in a separated testing room under ~15 W light intensity. Tests were video-recorded using a DCR-SX21E video camera recorder (Sony, Tokyo, Japan) and later manually analysed with the H77 computer-based event recorder.

### Open field test

An open field test (OFT) was used to determine the general locomotor activity and exploratory behaviour of rats. Rats were individually placed in the centre of a square arena fenced by white, non-transparent plastic walls (100 cm × 100 cm × 60 cm box) with a floor divided into 10 cm × 10 cm squares. The rats were allowed to explore freely for 10 min. The arena was cleaned with water between tests.

### Forced swim test

The classical Porsolt forced swim test (FST), commonly used in translational research on depression, was used to assess depression-like behaviour. This test is based on repeated exposure to an inescapable swim, which is repeated under the same test conditions after a 24 h interval. During the second test, animals typically spend a longer time floating and this is regarded as being the most sensitive sign of ‘behavioural despair’. The re-exposure of an animal to the originally presented adverse situation is intended to produce a state of learned helplessness, a behavioural hallmark of depression [21].

In our study, rats were placed in a cylindrical container (height 60 cm, diameter 14 cm) filled with tap water (25 ± 1°C). The pre-test period lasted for 15 min. The procedure was repeated 24 h later for a 5 min test session. Rats were fully dried and cylinders were filled up with fresh water after each test. The time rats spent floating (no movement except ones needed to keep the head over the water), struggling (vigorous limb movement, forelimbs break the surface of water, attempting to climb up on the cylinder) and swimming (coordinated movement with all four limbs, limbs are under the water) was recorded.

### Radiochemistry and in vivo imaging

To assess cerebrovascular blood flow and microglia activation, single-photon emission computed tomography (SPECT)–MRI imaging was carried out on anaesthetised rats. Before acquisition, an i.p. injection of 14 mg/kg potassium perchlorate (Sigma-Aldrich) was given followed by i.v. administration of 9.35 ± 2.00 MBq of ^125^I-labelled 6-chloro-2-(4′-iodophenyl)-3-(*N*,*N*-methylethyl)imidazo[1,2-a]pyridine-3-acetamide ([^125^I]CLINME) (Progressio, Budapest, Hungary) and, 30 min later, 135.86 ± 6.84 MBq of ^99m^Tc-labelled hexamethylpropyleneamine oxime ([^99m^Tc]HMPAO) (Medi-Radiopharma, Erd, Hungary). MRI acquisitions were performed immediately following the [^99m^Tc]HMPAO injection, using a nanoScan 1 T MRI system (Mediso, Budapest, Hungary). A T1-weighted GRE 3D sequence was used (460 μm in-plane resolution, slice thickness 500 μm, TR/TE 12/2 ms, TD 20 μs and four excitations resulting in a 9.5 min acquisition).

The SPECT–CT measurements were performed on a NanoSPECT/CT PLUS machine (Mediso) and results were quantified in units (0.33 mm, isovoxels) of radioactivity measured in MBq/ml. An atlas-based method (ventricles, cerebellum, cortex, hippocampus, striatum, bulbus olfactorius and one volume of interest [VOI] containing the whole brain) was used to segment the brain during MRI measurements.

For [^99m^Tc]HMPAO, the standardised uptake values (SUVs) of each segmented brain area were reported. For [^125^I]CLINME, the ratio of tissue activity to arterial blood activity was determined in every VOI using an image-based arterial blood activity concentration measurement. This value was normalised to the same area’s [^99m^Tc]HMPAO SUV as perfusion compensation. Image analysis of 3D SPECT VOIs was performed with VivoQuant 1.22 patch2 software (InviCRO, Boston, MA, USA).

### Fluorescence immunohistochemistry

Free-floating brain sections (25 μm thick) were either subjected to heat-induced epitope retrieval or simply blocked with 5–10% normal donkey serum and incubated overnight at 4°C with the appropriate mixture of primary antibodies. For visualisation, secondary antibodies labelled with AlexaFluor 488, 647 or 594 were used. Details of antibodies are listed in ESM Tables 1 and 2.

Images were captured with an Ni-E C2+ (Nikon, Tokyo, Japan) confocal microscope and image processing was performed using the NIKON NIS Elements Viewer 4.20 software (Auroscience, Budapest, Hungary). Quantitative analysis was carried out on three randomly selected fields of view within the region of interest for each brain section on 3-3 anatomically defined coronal sections.

### Western blot analysis

All reagents for western blot were purchased from Bio-Rad Laboratories (Hercules, CA, USA). Hippocampal samples were electrophoretically resolved, transferred and membranes were immunoblotted with specific primary antibodies. Horseradish peroxidase-conjugated secondary antibodies were used for chemiluminescence detection by Luminata Forte (Millipore Corporation, Billerica, MA, USA). Details of antibodies are listed in ESM Tables 3 and 4.

Densitometric analysis of bands was performed using Quantity One Analysis 4.6.6. software (Versadoc; Bio-Rad Laboratories). After background subtraction, integrated optical densities of bands of interest were factored for Ponceau S staining to correct for variations in total protein loading. Each blot was normalised to an internal control so that bands on separate blots could be compared.

### Real-time quantitative RT-PCR

RNA was extracted using the Total RNA Mini Kit (Geneaid Biotech, New Taipei City, Taiwan), reverse-transcribed by SuperScript III and the samples were diluted to 250 ng/μl (Life Technologies, Carlsbad, CA, USA). Expression levels *Il1a*, *Il6*, *Tnf*, *Bcl2*, *Bax* and 18S mRNA were determined by LightCycler 480 (Roche Diagnostics, Mannheim, Germany). Primer sequences are listed in ESM Table 5.

### Statistical analysis

Data were analysed in a blinded-fashion in behaviour tests and imaging experiments (SPECT-MRI and immunohistochemistry). Other experiments were not performed blind. Analysis was performed by one-way ANOVA followed by Holm–Sidak’s post hoc test using GraphPad Prism 6 software (GraphPad Software, San Diego, CA, USA) for multiple comparisons. For non-parametrical data the Kruskal–Wallis ANOVA on ranks followed by Dunn’s post hoc test was calculated. Pearson correlation was evaluated to reveal any interdependence of blood glucose or body weight and floating. Results are presented as means ± SD. *p* < 0.05 was considered as significant.

## Results

### Losartan ameliorates depression-like behaviour in diabetic rats

In the OFT, both vehicle- and losartan-treated diabetic rats performed fewer grid crossings, indicating that the general physical condition of these rats was worse than that of the non-diabetic control group (Fig. 1a). We investigated whether diabetes is associated with depressive behaviour in this experimental model. During the FST, floating time was longer in vehicle-treated diabetic rats than in control rats, reflecting the development of behavioural despair, a sign of depression-like behaviour (Fig. 1b).

Treatment of diabetic rats with losartan decreased floating time and, in parallel, increased the total time of mobility (Fig. 1b, c). When different movement variables were measured separately, the time spent struggling (the most representative movement pattern for active behaviour) was significantly increased by losartan treatment (Fig. 1d–f). These results suggest that losartan has a strong antidepressant effect. Since losartan did not influence locomotor activity in the OFT, the improved FST results can indeed be attributed to the antidepressant effect of losartan.

Diabetes led to decreased body weight gain and elevated blood glucose and fructosamine levels, none of which was improved by losartan (ESM Table 6). Neither body weight (*r* = −0.2601; *p* = 0.5339) nor blood glucose (*r* = −0.03226; *p* = 0.9396) influenced floating variables in diabetes.

### Diabetic rats show decreased cerebral perfusion, which is not altered by losartan

**Fig. 2:**
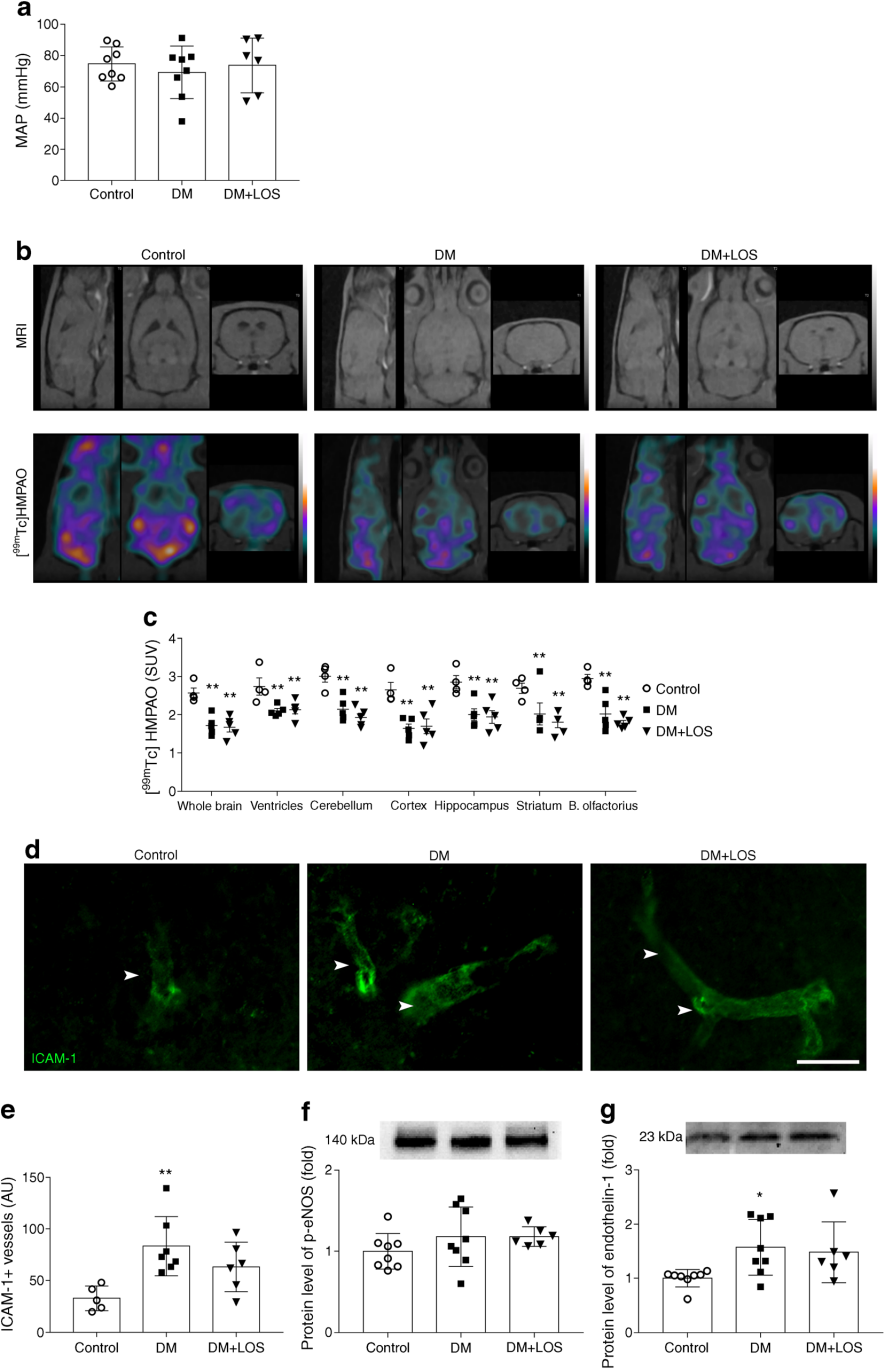
Decreased cerebral perfusion of diabetic rats is unaltered by losartan. (**a**) In control (non-diabetic) rats, vehicle-treated diabetic rats (DM) and losartan-treated diabetic rats (DM+LOS), mean arterial pressure was calculated. (**b**, **c**) Cerebral blood perfusion was measured by SPECT–MRI based on [^99mTc^]HMPAO uptake. Representative images from different sections of the whole brain. Colours represent the magnitude of [^99mTc^]HMPAO uptake (green, purple and yellow represent low, middle and high uptake, respectively) (**b**). Quantified data revealed significantly decreased perfusion in all investigated brain regions in DM and DM+LOS rats vs controls (**c**). (**d**, **e**) Cerebrovascular activation was assessed by ICAM-1 immunostaining in coronal brain sections of the dentate gyrus. Images are representative. Scale bar, 50 μm. (**d**) Integrated density of fluorescent images was determined by using Image J and expressed as arbitrary units (**e**). (**f**, **g**) Protein levels of p-eNOS (**f**) and endothelin-1 (**g**) in rat hippocampus were also evaluated (fold change vs control, which was set as 1). Ponceau S total protein staining was used as loading control (for a representative example of Ponceau S-stained membranes, see ESM Fig. 3a). Data are presented as mean ± SD (*n* = 4–5/group for imaging and *n* = 6–8/group for inflammatory markers and histology). *p < 0.05 and **p < 0.01 vs control rats. AU, arbitrary units; B. olfactorius, bulbus olfactorius

Cerebral perfusion, reported to be altered in experimental models of diabetes [22], was measured to study the mechanisms through which losartan may exert its actions on depressive behaviour. Since MAP remained unaltered in all groups (Fig. 2a), cerebral perfusion was studied using [^99m^Tc]HMPAO [23].

[^99m^Tc]HMPAO uptake was reduced in diabetic rats in every segmented brain region, indicating reduced cerebral perfusion. Losartan treatment did not change tracer uptake, suggesting unaltered cerebral blood flow (Fig. 2b, c).

Intercellular adhesion molecule 1 (ICAM-1) is a widely used marker of cerebrovascular activation [24]. Increased ICAM-1 immunostaining was observed in diabetic rats vs control rats and this increased level was not significantly reduced by losartan (Fig. 2d, e).

Endothelial nitric oxide synthase (eNOS) and endothelin-1 are both major regulators of cerebral blood flow. Phosphorylated eNOS (Ser1177; p-eNOS) levels did not differ between all groups of rats (Fig. 2f). Endothelin-1 protein level was higher in the hippocampus of diabetic rats and was not lowered by losartan treatment (Fig. 2g).

### Losartan ameliorates diabetes-induced neuroinflammation

**Fig. 3:**
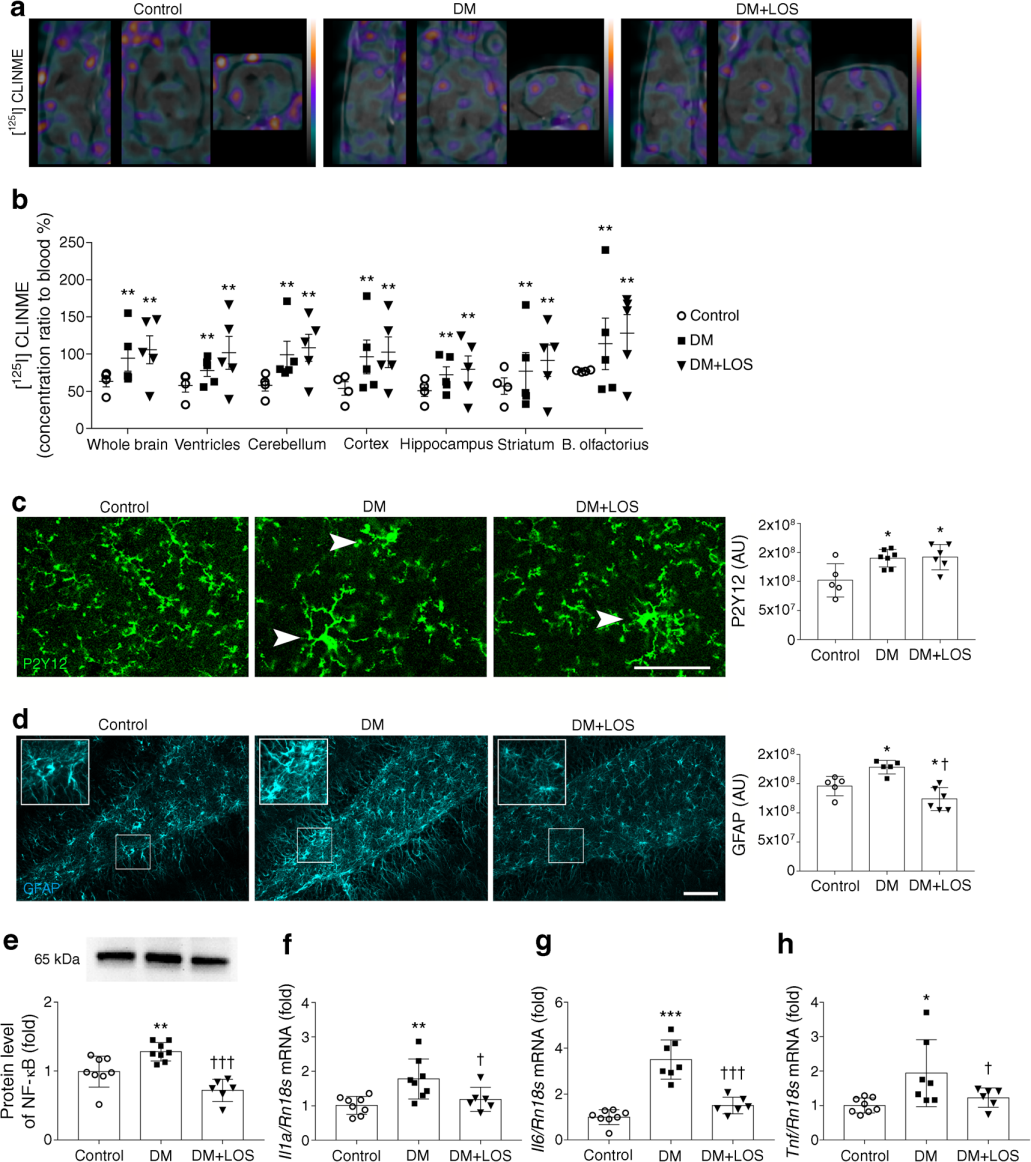
Losartan mitigates proinflammatory cytokine responses in diabetic rats. Neuroinflammation was assessed by various methods in control (non-diabetic), vehicle-treated diabetic rats (DM) and losartan-treated diabetic rats (DM+LOS). (**a**, **b**) Microglia activation was measured indirectly by SPECT–MRI based on [^125^I]CLINME uptake. (**a**) Representative images from different sections of the whole brain. Colours represent the magnitude of [^125^I]CLINME uptake (green, purple and yellow represent low, middle and high uptake, respectively) (**b**) Quantified data revealed significantly increased CLINME uptake in all investigated brain regions in DM and DM+LOS rats. (**c**) Microglial activation was assessed by P2Y12 immunostaining. Arrowheads showing P2Y12-positive microglia with thicker cell bodies and a partial withdrawal of processes (indicative of higher level of microglial activity). Scale bar, 50 μm. Quantification was based on unbiased densitometric analysis of P2Y12 signals in coronal brain sections of the dentate gyrus. (**d**) GFAP immunostaining showing astrocyte responses to diabetes and losartan and unbiased densitometric analysis showing reduced GFAP levels in coronal brain sections of the dentate gyrus in the DM+LOS group compared with the DM group (and control group). Scale bar, 100 μm. Due to technical problems (inappropriate immunostaining), 5/7 rats were analysed in the DM group. In (**c**) and (**d**), images are representative; integrated density of fluorescent images was determined by using Image J software. (**e**–**h**) Elevated protein level of NF-κB and mRNA expression of *Il1a*, *Il6* and *Tnf* were measured in the vehicle-treated diabetic rats; these effect was abolished by losartan treatment. Data are presented as fold change vs control, which was set as 1. Ponceau S total protein staining was used as loading control (for a representative example of Ponceau S-stained membranes, see ESM Fig. 3a.). *Il1a*, *Il6* and *Tnf* mRNA expression levels were normalised to *Rn18s* expression. For *Il6* and *Tnf*, due to technical problems (inappropriate RNA isolation and PCR), 7/8 rats were analysed in the vehicle-treated diabetic group. Data are presented as mean ± SD (*n* = 4–6/group for imaging and *n* = 6–8/group elsewhere). *p < 0.05, **p < 0.01 and ***p < 0.001 vs control rats; ^†^*p* < 0.05 and ^†††^*p* < 0.001 vs DM. AU, arbitrary units; B. olfactorius, bulbus olfactorius

Next, we investigated whether altered glial responses may explain the antidepressant effects induced by losartan. Activation of microglia, as assessed by translocator protein ligand [^125^I]CLINME using SPECT imaging in vivo, was increased in diabetic rats compared with control rats (Fig. 3a, b); this increase was not altered by losartan treatment. A similar pattern was observed in every segmented brain region (ventricles, cerebellum, cerebral cortex, hippocampus, striatum and bulbus olfactorius) (Fig. 3b).

Microglial activation [25] was also determined by co-staining with two independent markers, P2Y12 (Fig. 3c) and ionised calcium-binding adaptor molecule 1 (Iba1) (ESM Fig. 2). Diabetes was associated with an increased number of activated microglia (cells with thickened cell body, reduced number of processes and increased levels of reporter proteins, as revealed by both blinded cell counting and unbiased integrated density analysis). Diabetes-associated microglia activation was not altered by losartan treatment (Fig. 3c) and the total number of microglia remained unchanged as well (data not shown).

Analysis of glial fibrillary acidic protein (GFAP)-positive astrocytes, however, revealed that GFAP levels (increased in diabetic rats) were decreased by losartan (Fig. 3d), suggesting that losartan treatment may directly or indirectly influence astrocyte responses.

Since inflammatory responses in the brain are regulated via bidirectional astrocyte–microglia interactions [26], we next investigated how proinflammatory cytokine expression and signalling were altered by diabetes and/or losartan. In fact, increased expression of *Il1a*, *Il6* and *Tnf* proinflammatory cytokine mRNAs was detected in the hippocampus of diabetic rats (Fig. 3f–h). This was associated with elevated NF-κB protein levels (Fig. 3e), a key transcription factor involved in IL-1 and TNF signalling [27]. Losartan treatment significantly decreased NF-κB protein as well as *Il1a*, *Il6* and *Tnf* mRNA levels (Fig. 3e–h).

### Losartan induces the production of both BDNF forms

BDNF is synthesised as proBDNF, which is subsequently cleaved to mBDNF by intracellular (e.g. furin) or extracellular (e.g. matrix metalloproteinases [MMPs], serine protease plasmin) enzymes. Activation of the serine protease plasmin requires proteolytic cleavage by tissue plasminogen activator (TPA).

**Fig. 4:**
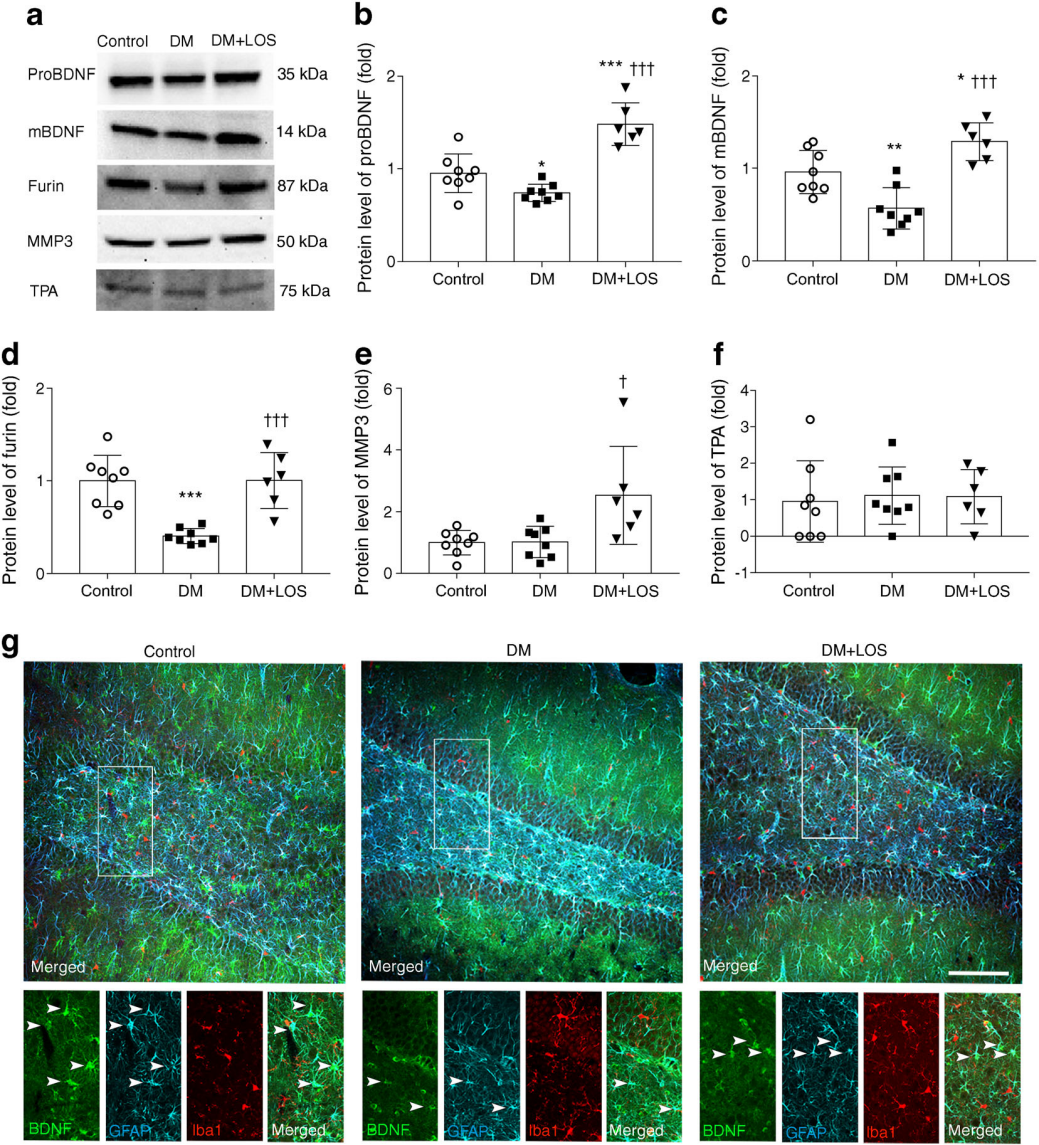
BDNF production and localisation is normalised by losartan in diabetic rats. (**a**–**f**) Representative blot (**a**) and quantification of protein levels of proBDNF (**b**), mBDNF (**c**) and cleavage enzymes (**d**–**f**) in the hippocampus of control (non-diabetic) rats, vehicle-treated diabetic rats (DM) and losartan-treated diabetic rats (DM+LOS). Data are presented as fold change vs control, which was set as 1. Ponceau S total protein staining was used as loading control (for a representative example of Ponceau S-stained membranes, see ESM Fig. 3b). (**g**) Triple immunofluorescence staining showing BDNF, GFAP-positive astrocytes and Iba1-positive microglia in the brain. Arrowheads in inserts indicate astrocytes with BDNF immunopositivity. Representative images of coronal brain sections of the dentate gyrus from three rats (representing each group) are shown. Scale bar, 200 μm. Data are presented as mean ± SD (*n* = 6–8/group). *p < 0.05, **p < 0.01 and ***p < 0.001 vs control rats; ^†^*p* < 0.05 and ^†††^*p* < 0.001 vs DM

Diabetes led to reduced proBDNF and mBDNF levels and this reduction was fully reversed by losartan treatment (Fig. 4a–c). Losartan also reversed diabetes-induced reduction in furin levels (Fig. 4a, d). Losartan increased MMP3 protein levels in diabetic rats vs control and vehicle-treated diabetic rats (Fig. 4a, e). TPA levels were similar in all groups (Fig. 4a, f). These results suggest that losartan may facilitate BDNF maturation in the hippocampi of diabetic rats.

BDNF, astrocytes (GFAP) and microglia (Iba1) were visualised by triple immunohistochemistry staining in the dentate gyrus of the hippocampus to identify the cell types responsible for BDNF production. The BDNF signal mostly colocalised with astrocytes and, in accordance with decreased BDNF protein levels, BDNF-positive astrocytes were less numerous in diabetic rats (Fig. 4g).

### Effect of losartan on hippocampal BDNF signalling

**Fig. 5:**
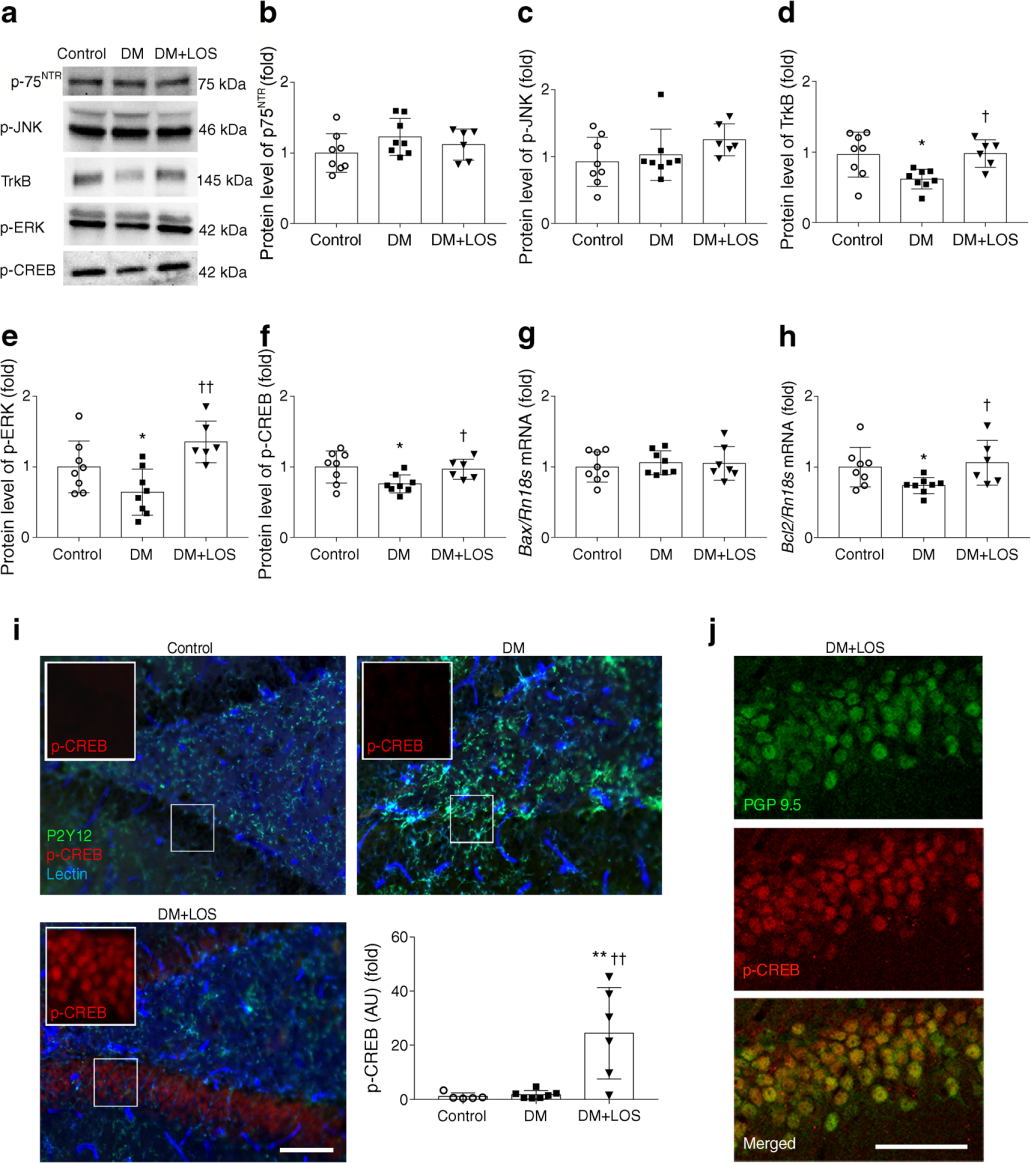
Losartan alters neuronal responses via mBDNF–TrkB signalling. (**a–f**) Representative blot and protein levels of p75^NTR^ (**b**), p-JNK (**c**), TrkB (**d**), p-ERK (**e**) and p-CREB (**f**), in the hippocampus of control, diabetic (DM) and losartan-treated diabetic (DM+LOS) rats. Ponceau S total protein staining was used as loading control (for a representative example of Ponceau S-stained membranes, see ESM Fig. 3b). (**g**, **h**) mRNA expression levels of *Bax* (**g**) and *Bcl2* (**h**) in the hippocampus. mRNA expression of *Bax* and *Bcl2* was normalised to *Rn18s* mRNA expression. In (**a**–**h**), Data are expressed as fold change vs control, which was set as 1. (**i**) Immunofluorescence images showing a marked upregulation of p-CREB in the hippocampal dentate gyrus in response to treatment with losartan. Integrated density of fluorescent images was determined using Image J software. Densitometric analysis showed a significant difference in p-CREB levels in DM+LOS compared with control and DM. No colocalisation of p-CREB with P2Y12-positive microglia (green) or blood vessels identified by tomato lectin (blue) can be seen. Scale bar, 100 μm. (**j**) p-CREB staining shows colocalisation with neurons identified by the pan-neuronal marker protein gene product 9.5 (PGP 9.5). Scale bar, 50 μm. In (**i**) and (**j**), representative images of the dentate gyrus are shown as captured in coronal brain sections. . Data are presented as means ± SD (*n* = 6–8/group). *p < 0.05 and **p < 0.01 vs control rats; ^†^*p* < 0.05 and ^††^*p* < 0.01 vs DM. AU, arbitrary units

ProBDNF mediates neuronal apoptosis and decreases synaptic plasticity via p75^NTR^. Levels of p75^NTR^ and phosphorylated c-Jun N-terminal kinase (JNK) proteins and pro-apoptotic *Bax* mRNA in rat hippocampus were unaffected by diabetes and losartan treatment (Fig. 5a–c, g). These results indicate that the proBDNF–p75^NTR^ pathway is not activated in the brains of diabetic rats.

mBDNF signalling mediates neuronal survival by binding to and activating the TrkB receptor. The protein level of TrkB, phosphorylated extracellular signal-regulated kinase (ERK) and phosphorylated cAMP response element-binding protein (CREB) and the mRNA level of anti-apoptotic *Bcl2* decreased in the hippocampus of diabetic rats (Fig. 5a, d–f, h). Losartan treatment of the diabetic rats markedly elevated TrkB, p-ERK and p-CREB protein levels as well as *Bcl2* mRNA expression levels.

p-CREB immunohistochemistry staining in the hippocampal dentate gyrus confirmed western blot results by showing that losartan treatment caused a marked upregulation of p-CREB, while diabetes alone had no effect (Fig. 5i). p-CREB colocalised with neurons labelled with the pan-neuronal marker protein gene product 9.5 (PGP 9.5) but not with P2Y12-positive microglia or blood vessels identified by tomato lectin, indicating that the changes seen in p-CREB levels are due to altered activity of hippocampal neurons (Fig. 5i, j). Thus, losartan interferes with the detrimental effects of diabetes on BDNF signalling in the brain, linking diabetes-induced depression with neuroinflammation and the potential role of BDNF in this experimental model.

## Discussion

The possible link between diabetes and depression is more than 300 years old and is a re-emerging paradigm. ARBs are a widely used clinical option for treatment of individuals with various diabetes-associated complications. Furthermore, clinical observations of the past decade unexpectedly revealed that ARB-treated hypertensive individuals have a lower requirement for antidepressants [28, 29]. In addition, ARBs improved interpersonal sensitivity and depression scores of individuals with type 2 diabetes [15]. However, the molecular mechanisms underlying these observations remain unexplored.

To test the hypothesis that ARBs may exert an antidepressant effect, we examined the effect of losartan in a rat STZ-induced experimental diabetes model. We showed for the first time that the depressive symptoms of diabetic rats can be effectively minimised by losartan treatment and that the antidepressant effect of losartan is independent of blood glucose or body weight.

Cerebrovascular abnormalities are common in diabetes and contribute to the development of depression [30, 31]. In addition, RAAS is a central regulator of cerebral perfusion and angiotensin II is the main effector causing local vasoconstriction. Given the known associations between cerebrovascular pathophysiology and depression (termed frequently as the ‘vascular hypothesis’ [32]), we first investigated whether alterations in cerebral perfusion might be regulated by the actions of losartan in diabetic rats. Impaired cerebral perfusion was verified by a remarkable decline in cerebral relative [^99m^Tc]HMPAO uptake in diabetic rats and this decline was not improved by losartan. We found that phosphorylated eNOS levels remained unchanged while endothelin-1 levels increased in diabetic brains, in parallel with elevated hippocampal ICAM-1 immunopositivity. These findings indicated vascular activation, although the reduction in these variables brought about by losartan did not reach statistical significance. Thus, our results suggest that the beneficial effects of losartan on behaviour may be independent of its direct vasoactive actions. This is in line with earlier observations showing mitigated neurological deficit by losartan without alterations in cerebral blood flow in a murine model of type 2 diabetes [33].

Neuroinflammation is associated with both diabetes and depression [34, 35], as indicated by the expression of proinflammatory mediators as well as microglia and astrocyte activation [36–38]. Increased [^125^I]CLINME-translocator protein uptake in diabetic rats suggested microglial activation, which was confirmed by immunohistochemistry, similar to that seen in other studies after 6 weeks of STZ-induced diabetes in the hippocampus [38]. Microglia and astrocyte activation was associated with the induction of an NF-κB-mediated inflammatory response with elevated *Il1a*, *Il6* and *Tnf* mRNA expression in diabetic rats, which was prevented by losartan. In line with this, losartan treatment decreased the response of astrocytes, but not of microglia, suggesting that diabetes-induced neuroinflammatory responses are modulated by losartan mainly via astrocytes. Previous studies in lipopolysaccharide-induced inflammation and hypertensive models showed that ARBs augment astrocyte and microglia activation [39, 40].

How mood disorders such as depression are influenced by inflammatory processes is not well understood. The causal role of proinflammatory mediators, namely IL-1 and TNF, has been widely proposed [41]. For example, IL-1-mediated actions may change neuronal network activity, the release of neurotransmitters, autonomic nervous system and hypothalamic–pituitary–adrenal axis responses and levels of growth factors among others [42, 43]. Recent studies indicate that neuroinflammatory processes may reduce BDNF expression [11, 12]. However, it remains unclear whether inflammation-induced changes in BDNF levels in the brain are associated with the formation of depression-related behaviours and whether this can be reversed therapeutically. Here, for the first time, we demonstrated changes in both forms of hippocampal BDNF in diabetes-associated depression. We also showed earlier that proBDNF and mBDNF levels were reduced in diabetes and were elevated by the antidepressant fluvoxamine [44]. Our present results indicate that diabetes-associated neuroinflammation, reduced BDNF levels and impaired BDNF signalling can be effectively reversed by an ARB. While microglia sense and respond to diabetes-induced effects, leading to long-lasting changes in their phenotype, this was not affected by losartan. On the contrary, losartan decreased astrocyte activation, reduced proinflammatory factors and restored BDNF signalling, all of which may contribute to the alleviation of depressive symptoms. Literary data also strengthen our point regarding the existence of this relationship. Diniz et al infused a TrkB receptor antagonist into the ventral hippocampus and prelimbic prefrontal cortex of rats and this prevented the antidepressant effect of losartan [45]. Furthermore, they subjected BDNF-haploinsufficient mice to FST and found that losartan decreased immobility time in wild-type rats but not in the rats with reduced BDNF levels. All these data support the direct link between losartan, BDNF and depression.

It is important to identify the exact molecular pathways through which impaired BDNF signalling may lead to the exacerbation of depressive symptoms in diabetes. Here, we found that proapoptotic p75^NTR^–JNK–B cell lymphoma 2-associated X protein (BAX) signalling is not activated, while the TrkB–ERK–CREB pathway is substantially impaired after 7 weeks of diabetes. However, the fact that despite losartan elevating proBDNF levels, proapoptotic p75^NTR^–JNK–BAX signalling was still suspended is rather interesting. It is plausible that losartan activated extra- and intracellular cleavage enzymes thereby promoting fast conversion of proBDNF to mBDNF. We confirmed this hypothesis by detecting increased furin and MMP3 levels in losartan-treated diabetic rats. Based on these results we postulate that proBDNF immediately transforms to mBDNF instead of activating its own receptor and downstream signalling.

The present study is the first to investigate the effect of ARB treatment on TrkB–ERK–CREB signalling in a model of diabetes. TrkB, p-ERK and p-CREB levels were decreased in the diabetic rat hippocampus, indicating that this pathway is repressed. Our results are in accordance with previous studies showing that TrkB and CREB activation is reduced in the hippocampus in diabetes [46, 47]. Losartan treatment elevated the TrkB, p-ERK and p-CREB levels, indicating that this pathway is activated and promotes neuronal survival. Immunohistochemical analysis also lent support to p-CREB levels being massively increased by losartan in the diabetic hippocampus.

CREB is known to trigger the expression of several neuroprotective proteins including B cell lymphoma 2 (BCL2) and BDNF [48]. Tanaka observed that p-CREB-positive neurons co-express BCL2 in the brain [49] and Ramirez et al showed that neurotrophins upregulated BCL2 expression [50]. In line with these observations, we show in the present study that losartan increased *Bcl2* expression and suggest that this may counteract the detrimental effects of diabetes-associated inflammatory changes in hippocampal neurons.

Based on these data we suggest that diabetes-induced neuroinflammation is, at least in part, responsible for decreased BDNF levels, which facilitate the development of depression-like behaviour. Our data also indicate that the upregulation of BDNF and p-CREB by ARBs may contribute to their neuroprotective effects in diabetic individuals and could be selectively targeted to alleviate some of the depressive symptoms associated with diabetes. Losartan also acts to restore the normal levels of BDNF via facilitating the conversion of proBDNF to mBDNF. In conclusion, our study suggests a novel potential of ARBs in diabetes-associated depression and may open up an additional therapeutic option for diabetic individuals.

## Electronic supplementary material

ESM (PDF 525 kb)

## Data Availability

Data are available on request from the authors.

## Abbreviations

ARB: Angiotensin receptor 1 blocker
BAX: BCL2-associated X protein
BCL2: B cell lymphoma 2
BDNF: Brain-derived neurotrophic factor
CREB: cAMP response element-binding protein
eNOS: Endothelial nitric oxide synthase
ERK: Extracellular signal-regulated kinase
FST: Forced swim test
GFAP: Glial fibrillary acidic protein
Iba1: Ionised calcium-binding adaptor molecule 1
ICAM-1: Intercellular adhesion molecule 1
[^125^I]CLINME: ^125^I-labelled 6-chloro-2-(4′-iodophenyl)-3-(*N*,*N*-methylethyl)imidazo[1,2-a]pyridine-3-acetamide
JNK: c-Jun N-terminal kinase
mBDNF: Mature brain-derived neurotrophic factor
MAP: Mean arterial pressure
MMP: Matrix metalloproteinase
OFT: Open field test
p75^NTR^: Neurotrophin receptor p75
proBDNF: Precursor brain-derived neurotrophic factor
RAAS: Renin–angiotensin–aldosterone system
SPECT: Single-photon emission computed tomography
SUV: Standardised uptake value
STZ: Streptozotocin
[^99m^Tc]HMPAO: ^99m^Tc-labelled hexamethylpropyleneamine oxime
TPA: Tissue plasminogen activator
TrkB: Tropomyosin receptor kinase B
VOI: Volume of interest
