# Supplementary material for: Novel therapeutic potential of angiotensin receptor 1 blockade in a rat model of diabetes-associated depression parallels altered BDNF signalling
Source: Diabetologia. 2019 May 3;62(8):1501–13. doi: 10.1007/s00125-019-4888-z (PMC6647092; doi:10.1007/s00125-019-4888-z)

ESM Tables

ESM Table 1. Fluorescent immunohistochemistry primary antibodies

Protein Target	Name of Antibody	Manufacturer	Catalog Number	Species Rised (Monoclonal or Polyclonal)	Dilution buffer	Dilution used
**BDNF**	anti-BDNF	Abcam, Cambridge, MA, USA	108319	rabbit-monoclonal	1x PBS, 20mM NA-azid, 0,3% Triton X-100	1:500
**GFAP**	anti-GFAP	Synaptic Systems, Goettingen, Germany	173006	chicken-polyclonal		
**ICAM-1**	anti- ICAM-1/CD54	R&D Systems, Minneapolis, MN, USA	#AF796	mouse-polyclonal		
**Iba1**	anti-AIF-1/Iba1	Novusbio, Littleton, CO, USA	NB100-1028	goat-polyclonal		
**IL-1α**	anti-IL-1 alpha /IL-1F1	R&D Systems, Minneapolis, MN, USA	AF-400-NA	goat-polyclonal		
**P2Y12**	anti-P2Y12	AnaSpec, Fermont, CA, USA	55043A	rabbit-polyclonal		
**p-CREB**	anti-pCREB	Cell Signaling Technology Inc., Danvers, MA, USA	87G3	goat-monoclonal		
**PGP9.5**	anti-PGP9.5	Abcam, Cambridge, MA, USA	72910	chicken-polyclonal		

ESM Table 2. Fluorescent immunohistochemistry secondary antibodies

Protein target	Name of Antibody	Manufacturer	Catalog Number	Species Rised	Dilution buffer	Dilution used
**Chicken IgG**	Alexa Fluor® 488 AffiniPure F(ab')₂ Fragment Donkey Anti-Chicken IgY (IgG)	Jackson ImmunoResearch Europe Ltd., Cambridgeshire, UK	703-546-155	donkey-polyclonal	1xPBS, 20mM NA-azid, 0,3% Triton X-100	1:200
**Goat IgG**	Donkey anti-Goat IgG (H+L) Cross-Adsorbed Secondary Antibody, Alexa Fluor 594	Thermo Fisher Scientific Waltham, MA USA	A11058	donkey-polyclonal		
**Mouse IgG**	Alexa Fluor® 647 AffiniPure Donkey Anti-Mouse IgG	Jackson ImmunoResearch Europe Ltd., Cambridgeshire, UK	715-605-150	donkey-polyclonal		
**Rabbit IgG**	Alexa Fluor® 488 AffiniPure F(ab')₂ Fragment Donkey Anti-Rabbit IgG	Jackson ImmunoResearch Europe Ltd., Cambridgeshire, UK	711-546-152	donkey-polyclonal		

ESM Table 3. Immunoblot primary antibodies

Protein Target	Name of Antibody	Manufacturer	Catalog Number	Species Rised (Monoclonal or Polyclonal)	Dilution buffer	Dilution used
**BDNF**	anti-BDNF	Abcam, Cambridge, MA, USA	108319	rabbit-monoclonal	1x TBS 0.05% wt/vol Tween 20	1:1000
**Endothelin-1**	anti-endothelin-1	Abcam, Cambridge, MA, USA	117757	rabbit-polyclonal	5% wt/vol non-fat milk, 1x TBS 0.05% wt/vol Tween 20	1:1000
**Furin**	anti-furin	Abcam, Cambridge, MA, USA	183495	rabbit-monoclonal	5% wt/vol non-fat milk, 1x TBS 0.05% wt/vol Tween 20	1:2000
**MMP3**	anti-MMP3	Abcam, Cambridge, MA, USA	52915	rabbit-monoclonal	5% wt/vol non-fat milk, 1x TBS 0.05% wt/vol Tween 20	1:1000
**NF-κB**	anti-NF-κB p65	Cell Signaling Technology Inc., Danvers, MA, USA	C22B4	rabbit-monoclonal	5% wt/vol bovine serum albumin, 1x TBS 0.05% wt/vol Tween 20	1:1000
**p75Ntr**	anti-p75NGF	Abcam, Cambridge, MA, USA	52987	rabbit-monoclonal	1x TBS 0.05% wt/vol Tween 20	1:500
**p-CREB**	anti-pCREB	Cell Signaling Technology Inc., Danvers, MA, USA	87G3	goat-monoclonal	1x TBS 0.05% wt/vol Tween 20	1:500
**p-eNOS**	anti-peNOS	Cell Signaling Technology Inc., Danvers, MA, USA	9571	rabbit-polyclonal	5% wt/vol bovine serum albumin, 1x TBS 0.05% wt/vol Tween 20	1:1000
**p-ERK**	anti-pERK	Santa Cruz Biotechnology, Santa Cruz CA, USA	sc-7383	mouse-monoclonal	1x TBS 0.05% wt/vol Tween 20	1:500
**p-JNK**	anti-pJNK	Cell Signaling Technology Inc., Danvers, MA, USA	9251	rabbit-polyclonal	5% wt/vol bovine serum albumin, 1x TBS 0.05% wt/vol Tween 20	1:1000
**TPA**	anti-TPA	Abcam, Cambridge, MA, USA	157469	rabbit-monoclonal	5% wt/vol non-fat milk, 1x TBS 0.05% wt/vol Tween 20	1:1000
**TrkB**	anti-TrkB	Santa Cruz Biotechnology, Santa Cruz CA, USA	sc-8316	rabbit-polyclonal	1x TBS 0.05% wt/vol Tween 20	1:500

ESM Table 4. Immunoblot secondary antibodies

Protein target	Name of Antibody	Manufacturer	Catalog Number	Species Rised	Dilution buffer	Dilution used
**Goat IgG**	Anti-goat IgG HRP-linked Antibody	Santa Cruz Biotechnology, Santa Cruz CA, USA	sc-2020	donkey	1x TBS 0.05% wt/vol Tween 20	1:3000
**Mouse IgG**	Anti-mouse IgG HRP-linked Antibody	Cell Signaling Technology Inc., Danvers, MA, USA	7076	goat		
**Rabbit IgG**	Anti-mouse IgG HRP-linked Antibody	Cell Signaling Technology Inc., Danvers, MA, USA	7074	goat		

ESM Table 5. Sequence of forward and reverse primers for qRT-PCR

Gene	NCBI reference	Primer pairs	Product length
Il1a	NM_017019.1	forward: 5' TCTGCCATTGACCATCTGTCTCTG 3'<br>reverse: 5' ACCACCCGGCTCTCCTTGAA 3'	152 bp
Il6	NM_012589.2	forward: 5' GCCACTGCCTTCCCTACTTC 3'<br>reverse: 5' GCCATTGCACAACTCTTTTCTC 3'	153 bp
Tnf	NM_012675.3	forward: 5' GGGGCCACCACGCTCTTCTGT 3'<br>reverse: 5' CTCCGCTTGGTGGTTTGCTACGAC3'	180 bp
Bcl2	NM_016993.1	forward: 5' ATGGCGCAAGCCGGGAGAACAG 3'<br>reverse: 5' TGGCGACAAGGGGGCCGTAGAGG 3'	232 bp
Bax	NM_017059.2	forward: 5' AGCCGCCCCAGGACGCATCCA 3'<br>reverse: 5' CAGCCGCTCCCGGAGGAAGTCCAG 3'	299 bp
Rn18s	NR_046237.1	forward: 5' GCGGTCGGCGTCCCCCAACTTCTT 3'<br>reverse: 5' GCGCGTGCAGCCCCGGACATCTA 3'	105 bp

ESM Table 6. Metabolic parameters were measured in control, diabetic (DM) and losartan-treated diabetic (DM+LOS) rats. Data are presented as means±SDs (n=6-8/group). **p<0.01 or ***p<0.001 *vs.* C. LOS: losartan.

	Control	DM	DM+LOS
**Weight gain (Δg)**	223±32.4	79.4±45.4**	80.0±20.0**
**Non-fasting blood glucose (mmol/L)**	13.2±1.62	44.6±9.13**	43.2±8.09**
**Fructosamine (µmol/L)**	153±11.9	262±14.4***	249±18.2***

ESM Figures

ESM Figure 1. Study design. STZ: streptozotocin, LOS: losartan, SPECT: Single photon emission computed tomography, MRI: Magnetic Resonance Imaging, OFT: open field test; FST: forced swim test.

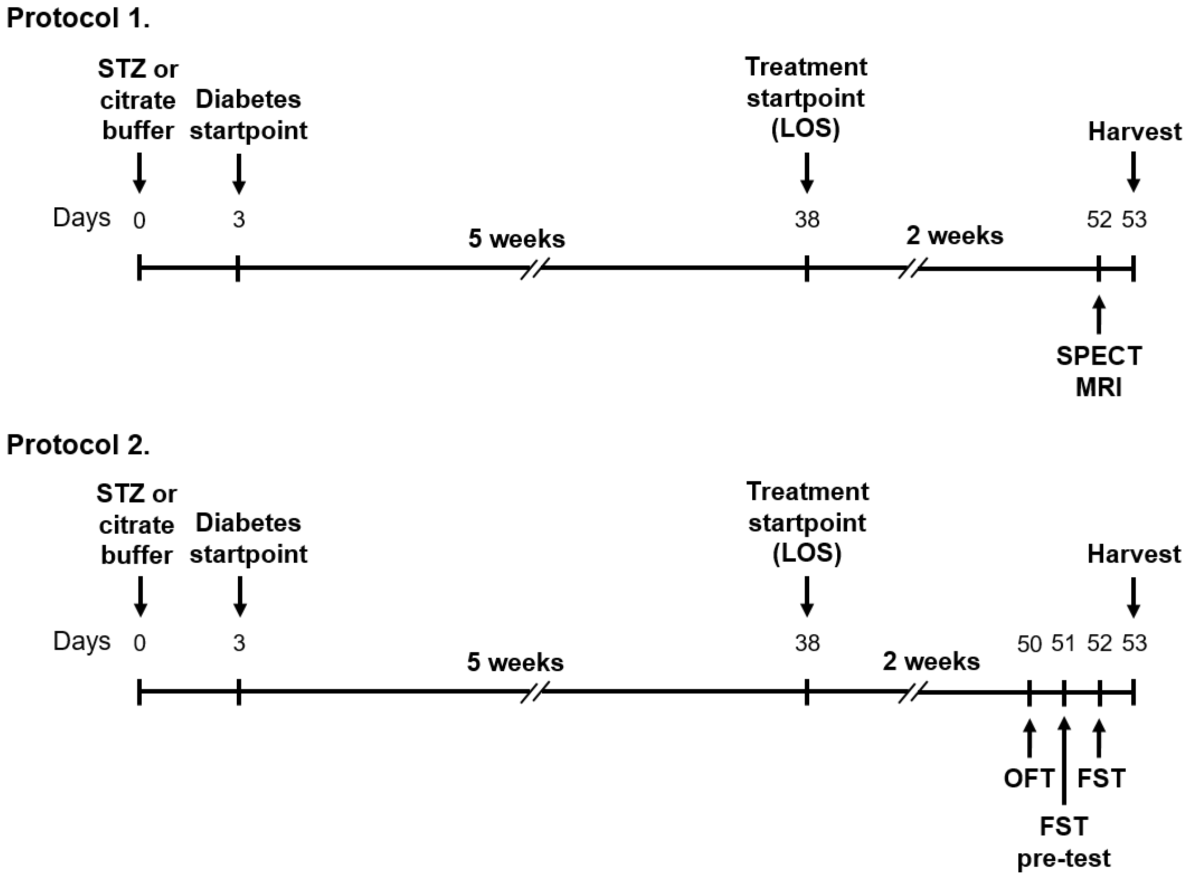

ESM Figure 2. Microglia were visualized by Iba1 to assess microglial activation in the hippocampus of control, diabetic (DM) and diabetic losartan-treated rats (DM+LOS). Arrowheads showing microglia with increased Iba1 expression, thicker cell bodies and a partial withdrawal of processes, indicative of higher level of microglial activity. Blinded evaluation is showing elevated numbers of activated microglia in the hippocampus of diabetic rats. Data are presented as means±SDs, (n=5-7/group). *p<0.05 *vs.* C. Scale bar: 50 µm.

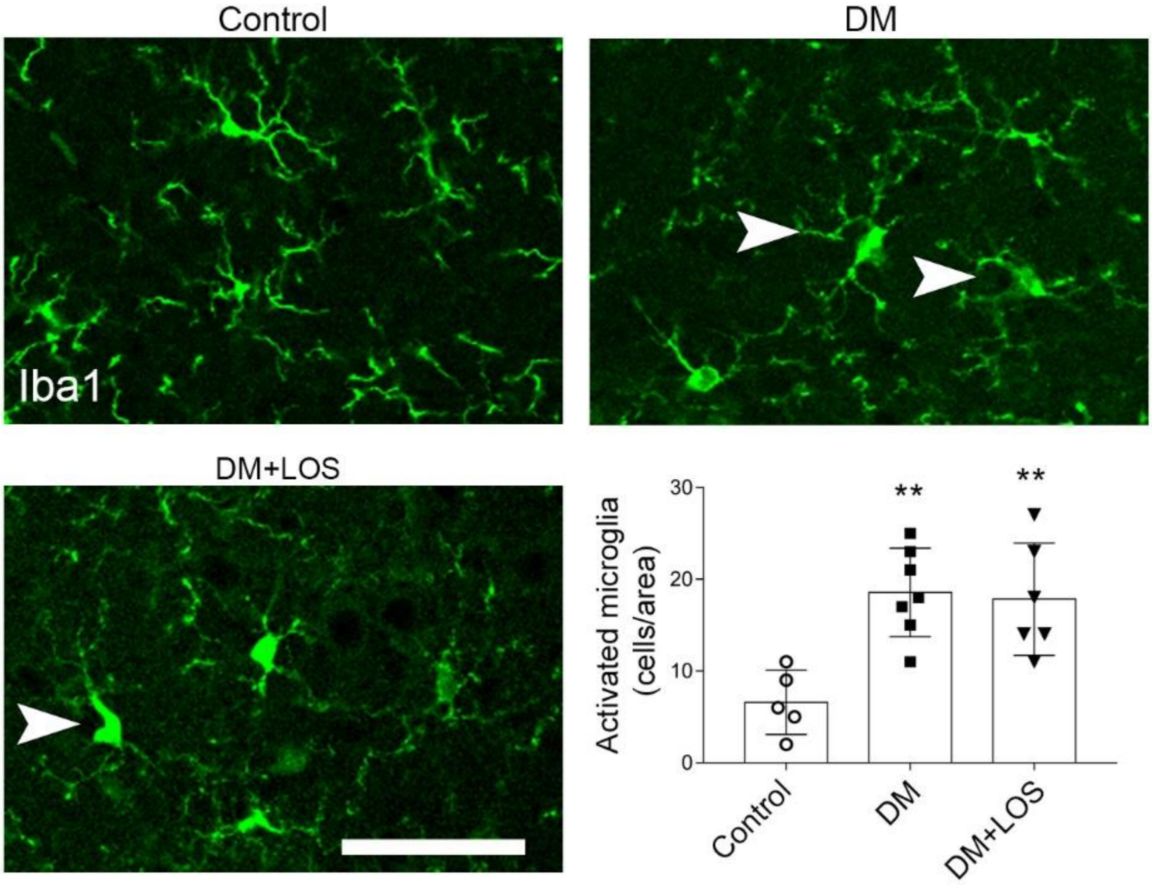

ESM Figure 3. Ponceau S-stained membranes of the representative samples. (a) Ponceau S-stained membranes of the representative examples of Figure 2 and 3. (b) Ponceau S-stained membranes of the representative examples of Figure 4. (c) Ponceau S-stained membranes of the representative examples of Figure 5. Black rectangles show the region of protein of interest. DM: diabetic group; DM+LOS: diabetic losartan-treated group.

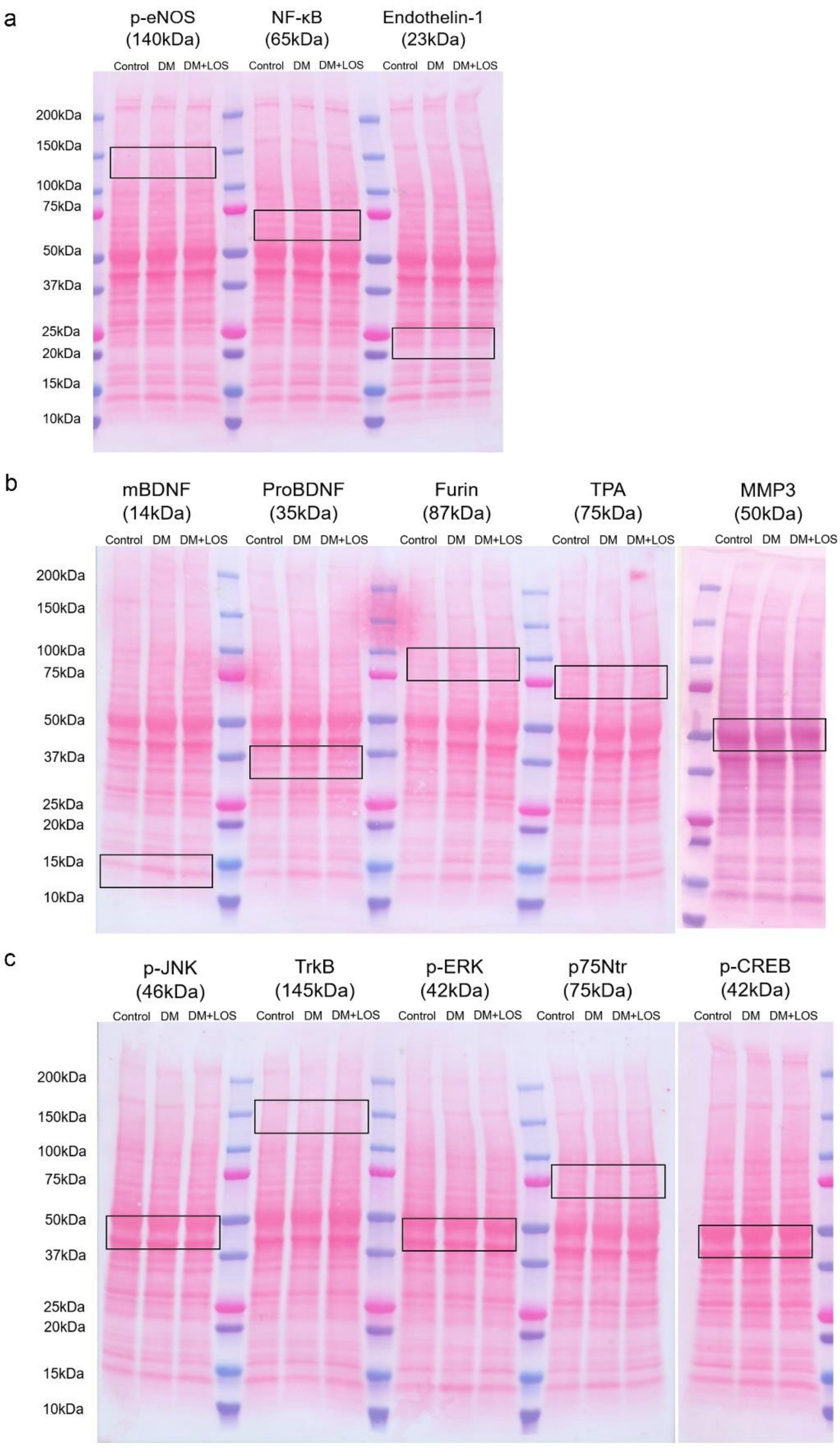

Supplement: Supplementary file 1 — (PDF 525 kb) [file 125_2019_4888_MOESM1_ESM.pdf]
